# Long-lived entanglement of molecules in magic-wavelength optical tweezers

**DOI:** 10.1038/s41586-024-08365-1

**Published:** 2025-01-15

**Authors:** Daniel K. Ruttley, Tom R. Hepworth, Alexander Guttridge, Simon L. Cornish

**Affiliations:** 1 Department of Physics, Durham University, Durham, United Kingdom; 2 Joint Quantum Centre Durham-Newcastle, Durham University, Durham, United Kingdom

**Keywords:** Atomic and molecular physics, Quantum physics

## Abstract

Realizing quantum control and entanglement of particles is crucial for advancing both quantum technologies and fundamental science. Substantial developments in this domain have been achieved in a variety of systems^1–5^. In this context, ultracold polar molecules offer new and unique opportunities because of their more complex internal structure associated with vibration and rotation, coupled with the existence of long-range interactions^6,7^. However, the same properties make molecules highly sensitive to their environment^8–10^, affecting their coherence and utility in some applications. Here we show that by engineering an exceptionally controlled environment using rotationally magic^11,12^ optical tweezers, we can achieve long-lived entanglement between pairs of molecules using detectable hertz-scale interactions. We prepare two-molecule Bell states with fidelity $0.92{4}_{-0.016}^{+0.013}$, limited by detectable leakage errors. When correcting for these errors, the fidelity is $0.97{6}_{-0.016}^{+0.014}$. We show that the second-scale entanglement lifetimes are limited solely by these errors, providing opportunities for research in quantum-enhanced metrology^7,13^, ultracold chemistry^14^ and the use of rotational states in quantum simulation, quantum computation and as quantum memories. The extension of precise quantum control to complex molecular systems will enable their additional degrees of freedom to be exploited across many domains of quantum science^15–17^.

## Abstract (web summary)

By engineering an exceptionally controlled environment using rotationally magic optical tweezers, long-lived entanglement between pairs of molecules using detectable hertz-scale interactions can be achieved.

## Main

Precise control of quantum states and the generation of entanglement are essential for unlocking the potential of quantum systems for developing new technologies and exploring fundamental science. Foundational work has focused on the quantum control of a variety of systems^1–5^, enabling many applications in quantum computing^18–20^, metrology^21–23^ and simulation^24^. Extending this control to more complex systems with more degrees of freedom, such as molecules, promises new advances in quantum metrology for fundamental physics^7,23^, the encoding of synthetic dimensions for quantum simulation^25^ and high-dimensional quantum computing^16,26^.

Ultracold polar molecules offer a rich internal structure associated with vibration and rotation, coupled with the existence of permanent electric dipole moments. These properties make molecules highly sensitive to a range of interesting phenomena^7,27,28^ and open up new prospects for studying ultracold chemistry^29,30^. In particular, the ladder of rotational states, with long radiative lifetimes, enables the storage of information and precise measurements over extended periods. Furthermore, neighbouring rotational states are connected through electric-dipole transition moments, giving rise to long-range interactions that can be precisely controlled with external fields. These properties may be exploited for a wide range of applications^31^, including high-dimensional quantum computation^6,16,17^ and quantum simulation^6,15,32^.

Recently, there has been rapid progress in the quantum control of molecules following the preparation of individual ultracold molecules in optical tweezers^33–37^. Pairs of molecules have been entangled^38–40^ and protocols have been developed to simultaneously read out multiple molecular states and to realize global and local single-particle gates^41,42^. Furthermore, mid-circuit detection and erasures of qubit errors have been demonstrated^43^. However, despite recent advances, molecules prepared in rotational-state superpositions remain highly sensitive to their trapping environment. To sustain single-particle coherence for ≳100 ms, rephasing pulse schemes are generally necessary^8–10^. This sensitivity restricts the interrogation time of individual molecules for precision metrology^7^ and reduces the lifetime of the generated entanglement^38^, thereby limiting their effectiveness as long-lived quantum memories and sensors.

In this work, we create an exceptionally controlled environment for ultracold molecules by using magic-wavelength optical tweezers that eliminate single-particle decoherence on experimental timescales. This enables us to entangle pairs of molecules with the highest reported fidelity to date, despite the hertz-scale interactions at our 2.8 μm particle spacing. Moreover, we demonstrate the entanglement of two molecules using direct microwave excitation, opening up the prospect of using shaped pulses to engineer entangling operations robust to experimental imperfections. Both approaches result in long-lived entanglement, which will enable quantum-enhanced second-scale metrology, quantum simulation and the encoding of quantum information within the rotational states of individually trapped molecules.

## Magic-wavelength optical tweezers

We begin by preparing molecules in a pristine environment that eliminates single-particle decoherence over typical experimental timescales. We assemble individually trapped ^87^Rb^133^Cs molecules in arrays of optical tweezers, producing and subsequently detecting pairs of molecules in about 7% of runs (Methods). To engineer long-range interactions, it is necessary to drive rotational transitions that enable pairs of molecules to interact through dipolar spin-exchange interactions^32^. Generally, rotational decoherence arises primarily from differential a.c. Stark shifts that cause the energies of rotational transitions to fluctuate as molecules sample different trapping intensities^10^.

To eliminate these deleterious light shifts, we trap the molecules in optical tweezers formed from light at a magic wavelength^11^ (Methods). This technique has previously been used in bulk-gas samples^12,44^ to achieve a rotational coherence time of 0.78(4) s (ref. ^12^). This method differs from earlier approaches for individually trapped molecules that used light at a magic polarization^8,9,45,46^. For these experiments, the longest reported coherence time, to our knowledge, was 93(7) ms (ref. ^8^), limited by second-order couplings between hyperfine states^10,47^. By using magic-wavelength light, we eliminate these couplings to first-order and second-order^47^.

**Fig. 1:**
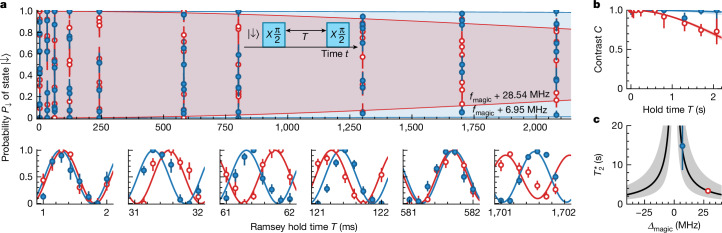
Multi-second rotational coherence for individually trapped molecules. **a**, Probability *P*_*↓*_ for a molecule to occupy the state |↓⟩ after the Ramsey sequence (inset). The bottom panels show detailed views of the top panel. The blue, filled points correspond to molecules trapped in a tweezer with frequency *f*_magic_ + 6.95(7) MHz and the red, empty points correspond to molecules trapped in a tweezer with frequency *f*_magic_ + 28.54(7) MHz. **b**, Ramsey fringe contrast as a function of the hold time *T* between the Ramsey pulses. The solid lines are a fit for a Gaussian noise model. **c**, The extracted ${T}_{2}^{* }$ times as a function of tweezer detuning *Δ*_magic_ from *f*_magic_. The solid line represents the expected behaviour with 0.7% intensity noise, whereas the shaded region shows the variation if this noise changes by a factor of two. Error bars in all plots show the 1*σ* confidence intervals and, on average, we use 199 experimental shots per data point. Source Data

We probe the rotational coherence of the molecules using a Ramsey interferometry sequence (Fig. 1a, inset), which does not contain any rephasing pulses. All molecules in our experiment begin in the rovibrational ground state |↓⟩, which we couple with the rotationally excited state |↑⟩ using microwave radiation (Methods). The pulse sequence contains two π/2 pulses, which drive the transition |↓⟩ → |↑⟩, with a hold time *T* between them. Figure 1a shows the relative probability *P*_*↓*_of molecules occupying the state |↓⟩ as *T* is varied. We use a multistate readout scheme to measure the internal state of each molecule^41^ and correct for state-preparation and molecule-loss errors with postselection (Methods). We probe for times *T* ≲ 2 s so that molecular interactions can be neglected (Methods). To measure some decoherence over this timescale, we set the detuning *Δ*_magic_ from *f*_magic_ of the first tweezer (blue, filled points) to 6.95(7) MHz and of the second tweezer (red, empty points) to 28.54(7) MHz. Here *f*_magic_ is the magic frequency at which the differential a.c. Stark shift between the states |↓⟩ and |↑⟩ is eliminated (Methods).

The dominant dephasing mechanism is shot-to-shot noise in the tweezer intensities. Figure 1b shows the Ramsey contrast *C* as a function of *T*. We fit *C* with a Gaussian noise model $C(T)={{\rm{e}}}^{-{(T/{T}_{2}^{* })}^{2}}$ from which we extract the Ramsey coherence times ${T}_{2}^{* }$ as 15(6) s and 3.3(2) s. Figure 1c shows ${T}_{2}^{* }$ as a function of *Δ*_magic_. We model the dephasing assuming Gaussian noise in tweezer intensities (Methods). The data are consistent with intensity noise (standard deviation) of 0.7% (solid line), which agrees with ex situ measurements of the tweezer powers. Our model predicts that, for ∣*Δ*_magic_∣ ≲ 0.5 MHz, ${T}_{2}^{* }$ due to trap dephasing exceeds a few minutes. Therefore, we have effectively eliminated rotational decoherence on relevant experimental timescales.

## Rabi spectroscopy of interacting molecules

**Fig. 2:**
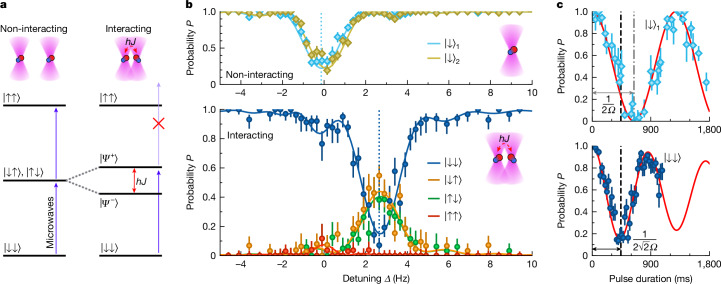
Microwave spectroscopy of a pair of interacting molecules. **a**, Eigenstates of the two-molecule system in the non-interacting (left) and interacting (right) cases. Interactions cause the single-excitation states |↓↑⟩ and |↑↓⟩ to couple to form two entangled states $| {\varPsi }^{\pm }\rangle \equiv (| \downarrow \uparrow \rangle \pm | \uparrow \downarrow \rangle )/\sqrt{2}$, which have an energy difference of *hJ*. We drive transitions between the eigenstates with microwaves. **b**, Microwave spectroscopy of single molecules (top) and pairs of molecules (bottom) using a square spectroscopy pulse of duration 441 ms and detuning *Δ* from the mean frequency of the single-molecule transitions. We show the probability of occupying different states using different colours (see text). **c**, Enhancement of the Rabi frequency by √2 when driving the two-molecule transition |↓↓⟩ → |*Ψ*^+^⟩ (bottom) compared with the single-molecule transition |↓⟩ → |↑⟩ (top). The detunings used when driving these transitions are shown by the dashed lines in **b**. The data in all panels are fitted simultaneously with a single set of free parameters; the solid lines show these fits. Error bars show the 1*σ* confidence intervals and, on average, we use 404 experimental shots per data point. Source Data

Figure 2a shows the eigenstates of a system of two molecules trapped in magic tweezers (Methods). Figure 2a (left) shows the non-interacting limit. The microwaves couple the ground state |↓↓⟩ to the degenerate states |↓↑⟩ and |↑↓⟩, which are coupled to the state |↑↑⟩. When the interaction between the molecules becomes significant, the singly excited states become coupled. Figure 2a (right) shows the resultant eigenstates, which include the two entangled states $| {\varPsi }^{\pm }\rangle \equiv (| \downarrow \uparrow \rangle \pm | \uparrow \downarrow \rangle )/\sqrt{2}$. The energy difference between these states is the energy *hJ* of the spin-exchange interaction. Microwaves can couple the symmetric states of the triplet manifold {|↓↓⟩, |*Ψ*^+^⟩, |↑↑⟩} such that the transition |↓↓⟩ → |*Ψ*^+^⟩ is allowed. By contrast, the antisymmetric singlet state |*Ψ*^*−*^⟩ is decoupled^48^.

We probe these energy levels with precision microwave spectroscopy. We form two near-magic tweezers that are separated by 2.78(5) *μ*m and use a square spectroscopy pulse of duration 441 ms, which drives the transition |↓⟩ → |↑⟩ with Rabi frequency *Ω* = 780(7) mHz. We study the non-interacting case (Fig. 2b, top) by postselecting on experimental runs in which only a single molecule was formed (Methods). The blue points show *P*_*↓*_ after the spectroscopy pulse for a molecule in the first tweezer and the gold points show *P*_*↓*_ for a molecule in the second tweezer. The microwave detuning *Δ* is relative to the mean frequency of the single-molecule transitions which differ by *δ* = 220(40) mHz.

When two molecules are present (Fig. 2, bottom), we can directly excite to the state |*Ψ*^+^⟩ when *Δ* ≈ *J*/2. Excitation out of the state |↓↓⟩ (blue) and into the state |↑↑⟩ (red) at zero detuning is suppressed because of the interaction shift in a rotational blockade effect^49^ analogous to Rydberg blockade. At *Δ* ≈ *J*/2, the slight asymmetry in the occupation of the states |↓↑⟩ (orange) and |↑↓⟩ (green) is because of the non-zero value of *δ* (Methods).

We verify that we drive a collective excitation by measuring an enhancement of the Rabi frequency for the transition |↓↓⟩ → |*Ψ*^+^⟩ compared with the single-molecule transition. When driving the transition |↓⟩ → |↑⟩ with a single molecule trapped in the first tweezer, we see oscillations at Rabi frequency *Ω* with a π-pulse duration of about 640 ms (Fig. 2c, top). By contrast, for the same microwave power, we drive the transition |↓↓⟩ → |*Ψ*^+^⟩ with enhanced Rabi frequency $\sqrt{2}\varOmega$ and a π pulse takes about 450 ms (Fig. 2c, bottom).

We model the dynamics of our system using a Monte Carlo approach (Methods), which allows us to fit the interaction strength *J*. We assume that shot-to-shot noise in *J* is such that, in each experimental iteration, *J* is sampled from a Gaussian distribution with mean ⟨*J*⟩ and standard deviation *σ*_*J*_. We fit ⟨*J*⟩ = 5.20(5) Hz and *σ*_*J*_ = 1.0(1) Hz. This is consistent with expected interaction strength in our system (Methods) and the solid lines in Fig. 2 show the dynamics predicted by this model.

## Spin-exchange entanglement

As a benchmark for the exceptional control that we realize in our experiment, we turn our focus to entangling pairs of molecules using hertz-scale interactions. This requires entangling operations over hundreds of milliseconds in which the dominant error is because of the molecular lifetimes.

**Fig. 3:**
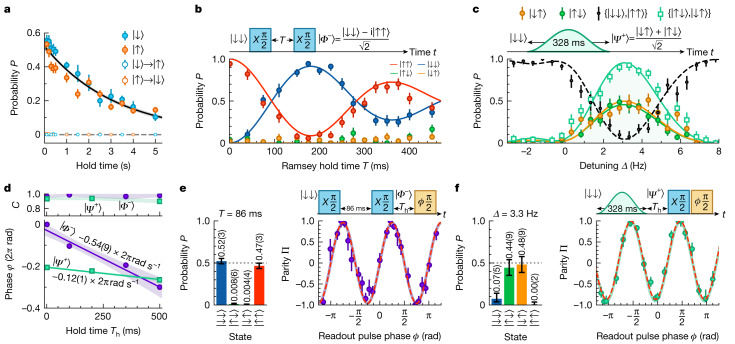
Preparation and characterization of long-lived molecular entangled states. **a**, Lifetime of single molecules in |↓⟩ (blue) and |↑⟩ (orange). We do not observe significant bit-flip errors (empty points). **b**, Molecule entanglement using spin exchange. We show the state probabilities *P* as a function of *T* in the Ramsey sequence shown. The colours are as in Fig. 2. **c**, Molecule entanglement with direct microwave excitation. We show the state probabilities *P*_*↓↓*_ + *P*_*↑↑*_ (black), *P*_*↓↑*_ + *P*_*↑↓*_ (empty green), *P*_*↑↓*_ (filled green) and *P*_*↓↑*_ (orange) as a function of detuning *Δ*. **d**, Long-lived entanglement for molecules in |*Φ*^−^⟩ (purple) and |*Ψ*^+^⟩ (green). Top, entanglement coherence ${\mathcal{C}}$ (correcting for leakage errors) after holding the entangled state for a time *T*_h_. The shaded regions are a guide to the eye. Bottom, phase *φ* of parity oscillations as a function of *T*_h_. The solid lines show linear fits and the shaded regions show the 1*σ* uncertainties of the fits. **e**, Measurement of the fidelity with which we entangle molecules with spin exchange using *T* = 86 ms. Left, state populations after the Ramsey sequence. Right, parity Π measured as a function of the phase of the readout pulse (see text) with a fit (dashed red line) and a model prediction (solid purple line, Methods). **f**, Measurement of the fidelity with which we entangle molecules with direct microwave excitation *Δ* = 3.3 Hz. Left, state populations after the microwave pulse. Right, measurement of the parity Π with a fit (dashed red line) and a model prediction (solid green line). Error bars in all plots show the 1*σ* confidence intervals and, on average, we use 486 experimental shots per data point. Source Data

The molecular lifetimes are limited by Raman scattering of the tweezer light. This causes leakage from the subspace {|↑⟩, |↓⟩} and apparent molecule loss due to the state specificity of our readout scheme^41^. Figure 3a shows the lifetimes of single molecules prepared in the states |↓⟩ (blue) and |↑⟩ (orange). For both states, the measured lifetime is 3.2(2) s. Crucially, this scattering does not cause bit-flip errors (that is, |↓⟩ $\nrightarrow$ |↑⟩ and |↑⟩ $\nrightarrow$ |↓⟩) as it is unlikely for a molecule to scatter back into the subspace {|↑⟩, |↓⟩}. This represents a perfect erasure error^50^, which we can detect.

First, we entangle pairs of molecules using resonant energy exchange. This method has been used to entangle molecules with interactions that are orders of magnitudes stronger than those in our system^38–40^. To generate this entanglement, we use the pulse scheme shown in Fig. 3b to prepare the molecules in rotational superpositions and wait to allow the resonant exchange of energy between the pair. Ideally, we transfer the state |↓↓⟩ to the state^38–40^

1 $$| \varPhi (T)\rangle =-\,{{\rm{e}}}^{-2{\rm{\pi }}{\rm{i}}\widetilde{T}}[\cos (2{\rm{\pi }}\widetilde{T})| \downarrow \downarrow \rangle -{\rm{i}}\sin (2{\rm{\pi }}\widetilde{T})| \uparrow \uparrow \rangle ],$$

where $\widetilde{T}\equiv JT/4$. This should result in spin-exchange oscillations between the states |↓↓⟩ and |↑↑⟩.

Figure 3b shows the result of applying this pulse sequence. The data points show the measured state populations and the solid lines show the results expected from our Monte Carlo model. As expected, we observe *P*_*↓↓*_ and *P*_*↑↑*_ oscillating with approximate frequency ⟨*J*⟩/2. The observed damping is caused by the non-zero value of *σ*_*J*_.

We expect to prepare molecules in the maximally entangled state $| {\varPhi }^{-}\rangle \equiv (| \downarrow \downarrow \rangle -{\rm{i}}| \uparrow \uparrow \rangle )/\sqrt{2}$ (ignoring the global phase) when *T* = 1/(2⟨*J*⟩). We find experimentally that we prepare |*Φ*^−^⟩ with the highest probability using *T* = 86(2) ms, slightly faster than the 96(1) ms predicted by our Monte Carlo model. The state populations after the entanglement sequence are *P*_*↓↓*_ = 0.52(3), *P*_*↑↑*_ = 0.47(3), and ${P}_{\uparrow \downarrow }+{P}_{\downarrow \uparrow }=0.01{2}_{-0.005}^{+0.009}$ (Fig. 3e, left).

We measure the fidelity of entanglement by incorporating a third readout pulse (Fig. 3e) to probe the two-particle coherence ${\mathcal{C}}$ (refs. ^38–40^). The duration of the hold before the readout pulse is *T*_h_ = 1 ms. We vary the phase *ϕ* of the readout pulse that causes oscillations in the parity $\Pi \equiv {P}_{\downarrow \downarrow }^{\phi }+{P}_{\uparrow \uparrow }^{\phi }-{P}_{\downarrow \uparrow }^{\phi }-{P}_{\uparrow \downarrow }^{\phi }$ of the form $\Pi (\phi )={\mathcal{C}}\,\sin (2\phi )$. Here, *P*^ϕ^ are the state populations measured after the readout pulse.

The data in Fig. 3e (right) show the measured behaviour of Π and the red dashed line shows a fit from which we extract ${\mathcal{C}}=0.96(2)$. The solid purple line shows the expected behaviour from our Monte Carlo model, which predicts ${\mathcal{C}}=0.95$. Our ability to perform state-specific readout of both |↓⟩ and |↑⟩ in a single experimental iteration allows us to eliminate most state-preparation and measurement errors using postselection^41^. Crucially, we can detect and disregard experimental runs with leakage errors to realize erasure qubits^51^. We can ignore the 5.3(3)% of runs in which there was a leakage error caused by Raman scattering during the 87 ms taken to entangle and probe the molecules. We extract entanglement fidelities $({P}_{\downarrow \downarrow }+{P}_{\uparrow \uparrow }+{\mathcal{C}})/2=0.97{6}_{-0.016}^{+0.014}$ with this correction and $({P}_{\downarrow \downarrow }+{P}_{\uparrow \uparrow }+{\mathcal{C}})/2=0.92{4}_{-0.016}^{+0.013}$ without this correction.

## Direct microwave entanglement

Our pristine environment eliminates the need for rephasing pulses, allowing us to explore the direct entanglement of molecules using microwaves. This enables applying quantum optimal control theory^52^ for designing robust entangling gates for molecules^48,53–55^. These gates are predicted to achieve fidelities of greater than 0.999 for ultracold molecules trapped in optical tweezers^48^.

Here we characterize the fidelity with which we can directly entangle molecules using a simple shaped pulse. We aim to drive the transition |↓↓⟩ → |*Ψ*^+^⟩ while minimizing off-resonant excitation to the state |↑↑⟩. We simulate this excitation with our Monte Carlo model to choose optimum pulse parameters (Methods). As a demonstration, we drive the transition with a Hann pulse of duration *τ* = 328 ms.

Figure 3c shows the measured state probabilities *P*_*↓↑*_ + *P*_*↑↓*_ (empty green) and *P*_*↓↓*_ + *P*_*↑↑*_ (black) as a function of *Δ*. The maximum value of *P*_*↓↑*_ + *P*_*↑↓*_ that we record experimentally is $0.9{3}_{-0.07}^{+0.04}$. The lines in Fig. 3c show the expected behaviour from our simulations of the system and the predicted maximum value of *P*_*↓↑*_ + *P*_*↑↓*_ (0.96) is within the experimental error.

We measure ${\mathcal{C}}$ with a similar method used to characterize the entanglement generated by spin exchange. Here, we set *Δ* = 3.3 Hz and the left panel of Fig. 3f shows the populations after the Hann pulse. For readout, we use two additional π/2 pulses on the transition |↓⟩ → |↑⟩ (Fig. 3f, top). These pulses occur *T*_h_ = 1 ms after the Hann pulse. The first readout pulse performs the transfer $| {\varPsi }^{+}\rangle \to (| \uparrow \uparrow \rangle +| \downarrow \downarrow \rangle )/(\sqrt{2}{\rm{i}})$ and we use the second pulse to measure Π as before. We vary the phase *ϕ* of the second readout pulse to obtain oscillations in Π (Fig. 3f, right).

From the data in Fig. 3f, we fit ${\mathcal{C}}=0.93(2)$ (dashed red line). The parity oscillation is slightly skewed towards Π = 1 because molecules that are not successfully entangled preferentially occupy the state |↓↓⟩. The measured coherence is within the error of that which we expect from our simulations (0.95, solid green line). From these measurements, we extract entanglement fidelities $0.9{3}_{-0.05}^{+0.03}$ when correcting and $0.7{6}_{-0.04}^{+0.03}$ when not correcting for the 19(1)% of runs in which a leakage error occurred during the Hann pulse.

## Entanglement lifetime

To use individually trapped ultracold molecules for applications in quantum metrology^7^ and quantum information processing^6^, it is highly desirable to produce long-lived entanglement. We investigate the coherence lifetime ${T}_{{\mathcal{C}}}$ of entangled pairs of molecules by varying the hold time *T*_h_ before applying the readout pulses. Figure 3d (top) shows the dependence of ${\mathcal{C}}$ on *T*_h_ for the states |*Φ*^−^⟩ (purple) and |*Ψ*^+^⟩ (green). For both states, we measure no significant change over 500 ms. This represents a notable improvement over previous work, in which ${T}_{{\mathcal{C}}}$ was limited by single-particle coherence times and rephasing pulses were required to achieve ${T}_{{\mathcal{C}}}$ of 61(3) ms (ref. ^38^). In our system, the 1.6(1) s lifetime of entanglement is limited solely by leakage errors caused by Raman scattering (Fig. 3a) and we can postselect to remove these errors with our detection scheme.

This long-lived entanglement paves the way for measuring sub-hertz energy shifts with quantum-enhanced metrology^7,13^. First, we consider the state |*Φ*^−^⟩. In the rotating frame, a global energy difference *ΔE* between the states |↓⟩ and |↑⟩ causes |*Φ*^−^⟩ to evolve in time *t* to the state $(| \downarrow \downarrow \rangle -{\rm{i}}{{\rm{e}}}^{-{\rm{i}}\varphi }| \uparrow \uparrow \rangle )/\sqrt{2}$, where *φ* = −2*ΔEt*/*ħ*. Here, the factor of 2 in the phase *φ* highlights the enhanced sensitivity of this state to global perturbations, which can be leveraged to achieve Heisenberg-limited precision^23^. We measure the rate of phase accumulation d*φ*/d*t* from the measurements of Π (Fig. 3d, bottom) and extract 2*ΔE*/*h* = 540(90) mHz. This reflects a detuning *ΔE*/*h* between the microwave field and the molecular transition frequency, allowing us to precisely measure the energy of the transition |↓⟩ → |↑⟩. By contrast, when we directly excite to the state |*Ψ*^+^⟩, we occupy an eigenstate of *H* that is within a decoherence-free subspace and is immune to collective dephasing^56^. The component states (|↓↑⟩ and |↑↓⟩) of |*Ψ*^+^⟩ accrue a relative phase only if the energy of the transition |↓⟩ → |↑⟩ varies inhomogeneously during *T*_h_, providing a sensitive probe to local perturbations. Moreover, encoding of quantum information in these states has been demonstrated to increase the lifetime by multiple orders of magnitude^56^, making these states attractive for realizing quantum memories. Any phase accrual partially transfers |*Ψ*^+^⟩ → |*Ψ*^−^⟩, which does not couple to microwave pulses, causing the measured value of ${\mathcal{C}}$ to decrease while preserving the phase *φ* of the parity oscillations. We detect no significant change in ${\mathcal{C}}$ over this timescale, and therefore conclude that local perturbations in the rotational splitting during *T*_h_ are sub-hertz.

## Outlook

We have realized long-lived entanglement between pairs of molecules. This required the engineering of a pristine environment that eliminates rotational decoherence on experimental timescales. Operating in this environment, we have prepared two-molecule Bell states using dipolar spin exchange and direct microwave excitation with fidelities $0.92{4}_{-0.016}^{+0.013}$ and $0.7{6}_{-0.04}^{+0.03}$, respectively, limited by detectable leakage errors. When correcting for these errors, these fidelities are $0.97{6}_{-0.016}^{+0.014}$ and $0.9{3}_{-0.05}^{+0.03}$. This represents the highest reported entanglement fidelity, to our knowledge, for individually trapped polar molecules to date and one of the first realizations of a two-molecule microwave gate. Furthermore, these methods prepare Bell states that are sensitive to either the global or local environment, realizing sensitive probes of different physical phenomena.

In the near term, the speed and fidelity of our Bell-state preparation may be improved by changing the confinement of the molecules to access smaller separations. For example, transferring the molecules into a magic-wavelength optical lattice should give access to sub-micrometre separations and increased molecular confinement, resulting in increased interaction strengths with reduced noise. These improvements will allow the implementation of high-fidelity two-molecule gates^48,57^ that entangle molecules on the millisecond timescale, while preserving the pristine environment and long-lived entanglement associated with magic-wavelength trapping.

Furthermore, our results show that there are no fundamental obstacles to using ultracold molecules for a wide range of applications in quantum science. The ability to prepare molecules in various Bell states opens up new avenues for studying quantum interference effects in ultracold chemistry^14^. Moreover, the deterministic preparation of molecules in a decoherence-free subspace paves the way for quantum-enhanced metrology^13^ and the use of long-lived rotational states as quantum memories within hybrid quantum systems^58–60^. Finally, our modelling suggests that second-scale coherence can be simultaneously achieved for multiple rotational transitions^11,12^; this will allow the ladder of molecular rotational states to be exploited as qudits^16^ or synthetic dimensions^15^.

## Methods

### Experimental apparatus

In our experimental apparatus^61,62^, we produce ultracold ^87^Rb^133^Cs (hereafter RbCs) molecules trapped in one-dimensional arrays of optical tweezers at wavelength 1,065.512 nm (hereafter 1,066 nm). The molecules are trapped inside an ultrahigh vacuum glass cell, with the tweezers formed by focusing light through a high numerical aperture objective lens placed before this cell. The molecules are formed by associating Rb and Cs atoms as described in ref. ^41^.

#### Magic-wavelength tweezers

**Extended Data Fig. 1:**
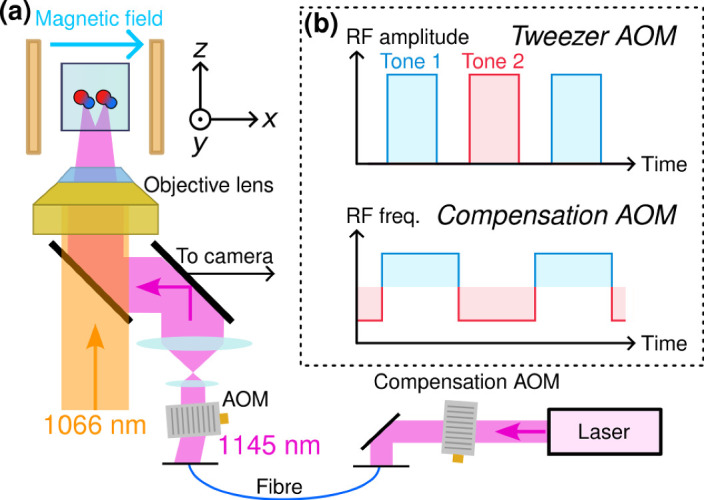
Generation of multiple magic-wavelength tweezers. (a) Simplified optical setup of the magic tweezers used in our experiment. We generate an 1,145 nm tweezer array by driving an AOM with multiple RF tones. An additional compensation AOM can be used to modulate the frequency of the input light. (b) Schematic of the modulation scheme used to generate two time-averaged tweezer traps with the same laser frequency. Upper: the RF amplitudes of the two frequency tones used to drive the tweezer AOM in order to generate two time-averaged traps. Lower: simultaneous switching of the RF frequency with which we drive the compensation AOM ensures that the light delivered to the molecules has the same frequency for both tweezers.

For the work presented here, we have added a set of tweezers at a magic wavelength of 1,145.31 nm. This light is in the vicinity of a weakly allowed electronic transition^11,44^ and eliminates the differential a.c. Stark shift *hΔα*_a.c._ (ref. ^63^) for the rotational transition |↓⟩ → |↑⟩. We determine the magic wavelength by measuring *hΔα*_a.c._ with a Ramsey procedure and setting the frequency of the traps so that *hΔα*_a.c._ is eliminated (Hepworth, T. R. et al., manuscript in preparation). In front of the objective lens, the polarization of the tweezers is parallel to the quantization axis set by the external magnetic field. The array of tweezers is created with an acousto-optic modulator (AOM) placed before the objective lens (Extended Data Fig. 1a). By applying multiple radio-frequency (RF) tones to the AOM, we form multiple diffracted beams to generate the tweezers. We dynamically switch and move the tweezers by changing the RF tones applied to the AOM to manipulate the trapped molecules mid-routine. By imaging Cs atoms trapped in the magic tweezers, we calibrate the change in tweezer position (at the focal plane) with the change in RF frequency applied to the AOM as 397(7) nm MHz^−1^.

We perform parametric heating measurements^64^ of Cs atoms trapped in the magic tweezers to characterize their 1/*e*^2^ beam waists. To do this, we modulate the intensity of the traps and measure a loss feature that occurs when the modulation frequency is twice that of the trap frequency. We assume the light in the focal plane is well described by a Gaussian beam and take the polarizability of the Cs atoms to be $918(3)\times 4{\rm{\pi }}{\varepsilon }_{0}{a}_{0}^{3}$ (ref. ^65^) to obtain the 1/*e*^2^ waist 1.76(4) μm.

For efficient transfer of molecules between different tweezer arrays, the tweezers must be well overlapped. We overlap the tweezers in the radial directions by imaging Cs atoms in both sets of tweezers and moving the magic tweezers until the positions of the atoms overlap. This enables us to overlap the centre of the tweezers to sub-micrometre accuracy. This method is much less sensitive to the overlap in the direction of tweezer-light propagation. We coarsely overlap the arrays in this direction by moving a lens in the expansion telescope of the 1,145 nm light so that atoms in both arrays are in focus on our imaging camera. We expect that there could be an alignment error of up to a few micrometres in this direction.

To transfer molecules between the two arrays, we start with the tweezers overlapped. We ramp up the power of the magic tweezers and then ramp down the power of the 1,066 nm array. During this step, the separation between neighbouring tweezers is approximately 4 μm. After this transfer, we switch off excess tweezers to discard the excess molecules. At the end of the experiment, we transfer the molecules back to the 1,066 nm array before disassociating them and reimaging their constituent atoms. During this process, we map the internal state of the molecule onto atomic position for multistate readout^41^.

To tune the dipolar interaction strength between molecules, we tune the separation of the molecules by chirping the frequency of the RF tones that generate their tweezers. For all the experiments presented in the main text, we move a pair of molecules symmetrically around their mean position to minimize the chance that a molecule is preferentially heated during the movement process.

#### Magic-frequency stabilization

In previous work trapping RbCs molecules in magic-wavelength traps^12^, the single-molecule coherence time was limited by the frequency stability of the trapping laser. The laser was stabilized to a cavity of finesse approximately 400, and a frequency stability (standard deviation) of 0.76 MHz was achieved.

For this work, we reference an external-cavity diode laser (ECDL; Toptica DL pro) at 1,145 nm to an ultralow expansion cavity (Stable Laser Systems) with a finesse of about 3.7 × 10^4^. We stabilize this laser with a fast feedback loop (Toptica FALC pro) and achieve a linewidth of around 5 kHz. To allow for future scaling to larger arrays, we source the trapping light from a vertical-external-cavity surface-emitting laser (Vexlum VALO), which provides up to 4 W of optical power. We stabilize the beat note between this laser and the ECDL. Feedback to the laser frequency is achieved using a piezo-electric actuator mounted on a mirror in the laser cavity. With stabilization, the standard deviation of the beat-note signal is 80(20) kHz. Therefore, we expect the frequency of the trapping light to be stable to within 80(20) kHz.

#### Tweezer-intensity noise

In Fig. 1c, we show the measured single-molecule coherence times ${T}_{2}^{* }$ as a function of the detuning *Δ*_magic_ of the tweezers from the magic frequency *f*_magic_. We model the effect of intensity noise in our experiment to understand the behaviour of ${T}_{2}^{* }$ with *Δ*_magic_ and briefly discuss that model here.

We determine *f*_magic_ and the sensitivity of the molecules to *Δ*_magic_ with a Ramsey procedure (Hepworth, T. R. et al., manuscript in preparation). The differential a.c. Stark shift *hΔα*_a.c._ is proportional to the power *P* of each tweezer and *Δ*_magic_. The scaling constant *k* = 923(3) mHz MHz^−1^ mW^−1^ relates these such that *Δα*_a.c._ = *kΔ*_magic_*P*. The power in each tweezer is measured before the objective lens; we estimate that the transmission from this location to the science cell is 0.48(1).

To model the intensity noise, we assume that there is Gaussian noise on *P* such that it is sampled from a Gaussian distribution with mean ⟨*P*⟩ and standard deviation *σ*_*P*_. For the measurement in Fig. 1, ⟨*P*⟩ = 0.36 mW. This noise is mapped to *Δα*_a.c._ with standard deviation *σ*_*α*_ = *kΔ*_magic_*σ*_*P*_. Therefore, the Ramsey contrast $C(T)=\exp [-{(2{\rm{\pi }}{\sigma }_{\alpha }T)}^{2}/2]\equiv \exp [-{(T/{T}_{2}^{* })}^{2}]$ (ref. ^66^). Hence, ${T}_{2}^{* }=1/(\sqrt{2}{\rm{\pi }}{\sigma }_{\alpha })$ and the solid line in Fig. 1c shows the predicted behaviour when *σ*_*P*_/⟨*P*⟩ = 0.7%.

#### Achieving magic trapping conditions for multiple tweezers

For the experiment in Fig. 1, we prepare single molecules in pairs of tweezers separated by 8.6(2) μm. They are generated using a frequency difference of *Δf* = 21.7 MHz between the two RF tones applied to the AOM and the power per tweezer is actively stabilized to ⟨*P*⟩ = 0.36 mW. Therefore, we expect *Δα*_a.c._ would be different by *δ* = *k*⟨*P*⟩*Δf* = 7.2 Hz. The data in Fig. 1a are fitted with a damped sinusoidal function with frequency *ν*. For the tweezer that is closer to *f*_magic_ (blue, filled points), we fit *ν* = 999.26(2) Hz, and for the tweezer that is further detuned (red, empty points), we fit *ν* = 992.49(1) Hz. This is a frequency difference of 6.77(3) Hz, approximately 6% smaller than expected.

For the experiments in Figs. 2 and 3, the tweezer separation is 2.78(5) μm. Each tweezer has a time-averaged power of ⟨*P*⟩ ~ 0.3 mW and is generated by RF tones with a frequency difference of *Δf* = 7.011 MHz. This difference in detuning from the magic frequency results in a difference in transition frequency between the two molecules of *δ* = *k*⟨*P*⟩*Δf* ~ 2 Hz.

To engineer the regime *δ* ≪ *J*, we minimize *δ* by minimizing *Δf* while maintaining the same tweezer separation. To do this, we modulate the tweezer intensities in antiphase at a frequency of 500 kHz with a duty cycle of 0.35. Simultaneously, we modulate the frequency of an RF tone applied to a compensation AOM so that, ideally, the light forming the two tweezers has an identical frequency. A schematic of the modulation scheme is shown in Extended Data Fig. 1b. The 500 kHz modulation frequency is far above any parametric resonances and we do not observe any change in the molecule-loss rate due to the modulation. We do not actively stabilize the tweezer intensity when operating in this regime. We have verified that this modulation does not affect single-molecule coherence by repeating measurements such as those in Fig. 2c (top) with and without this modulation. We attribute the non-zero value of *δ* reported in the main text to the non-zero decay time of tones in the amplifier that drives this compensation AOM.

In future, we plan to scale to larger molecule arrays with methods that will not require this compensation AOM. For example, by using a spatial light modulator to form the magic tweezers, as we do for the 1,066 nm tweezers^41^, all tweezers will have the same frequency. Alternatively, a pair of crossed acousto-optic deflectors could be used to create arrays of magic-wavelength tweezers with a constant frequency across the array^67^. Moreover, we note that all sites in a magic-wavelength (one-dimensional) optical lattice would have the same frequency.

#### Microwave excitation

In our experiment, we prepare RbCs molecules in the absolute internal ground state |↓⟩ = |*N* = 0, *M*_*N*_ = 0, *m*_Rb_ = 3/2, *m*_Cs_ = 7/2⟩. Here, *N* is the rotational quantum number, *M*_*N*_ is its projection, *m*_Rb_ is the projection of the nuclear spin of Rb and *m*_Cs_ is the projection of the nuclear spin of Cs. We couple this state to the excited rotational state |↑⟩ = |*N* = 1, *M*_*N*_ = 1, *m*_Rb_ = 3/2, *m*_Cs_ = 7/2⟩. Both of these states are stretched with maximum projections of angular momentum. In our experiment, the quantization axis is set by the externally applied magnetic field (about 181.7 G), which stays approximately constant for all science stages of the experiment.

The transition |↓⟩ → |↑⟩ is magnetically insensitive. The dominant contribution to the Zeeman shifts of the states |↓⟩ and |↑⟩ is associated with the projection of the nuclear spins. However, as these are both stretched states with the same *m*_Rb_ and *m*_Cs_, their nuclear-spin Zeeman shifts are equal. The rotational Zeeman effect is very small^68,69^, leading to a differential Zeeman shift of about 5 Hz G^−1^ × *h*. In our experiment, we stabilize the magnetic field to the approximately 10 mG level so that the differential shift does not vary from shot to shot. We expect that this magnetic field noise will limit single-molecule coherence times to the approximately 10 s level.

We drive the molecular transition |↓⟩ → |↑⟩ with microwaves radiated from a dipole Wi-Fi antenna mounted approximately 10 cm from the vacuum chamber. The frequency of the transition in free space (or in a perfectly magic tweezer) is 980.38559837(4) MHz (Hepworth, T. R. et al., manuscript in preparation). The resultant microwaves are not well polarized, so it would be possible to drive transitions to other rotational states. For this reason, we use Rabi frequencies ≲10 kHz such that off-resonant excitation to other states is negligible^41^ and each molecule can be considered a two-level system. For kilohertz-scale Rabi frequencies, we drive the antenna with an Agilent E4400B source and typically input a microwave power of about 0 dBm to the antenna. We vary the phase of this source when measuring the parity Π presented in Fig. 3. For hertz-scale Rabi frequencies, we use an Anritsu MG369xC source set to about −15 dBm with a further 55 dB of attenuation. We amplitude modulate this source with an arbitrary function generator (Tektronix AFG3022C) when using the Hann pulse for direct microwave entanglement. The sources are combined before the antenna with an RF switch (Minicircuits ZFSWA2R-63DR+) and are referenced to the same 10 MHz GPS signal to ideally maintain a constant, but arbitrary, relative phase. We attribute the observed d*φ*/d*t* for the state |*Ψ*^+^⟩ (Fig. 3d, bottom, green data points) to a slight phase drift between these microwave sources.

### Microwave pulse sequences

We probe single-molecule coherences and generate entanglement with spin exchange using the Ramsey pulse sequences shown in Figs. 1a and 3b, respectively. In both sequences, we apply two π/2 pulses on the single-molecule transition |↓⟩ → |↑⟩ with a hold time *T* between them. Both pulses have the same phase which we use to define the $\widehat{x}$-axis of the Bloch sphere. Neither of these sequences includes any rephasing pulses. The microwaves drive the transition with Rabi frequency *Ω* = 5.0(1) kHz.

For the measurement in Fig. 1, the first pulse prepares each molecule in the state $(| \downarrow \rangle +{\rm{i}}| \uparrow \rangle )/\sqrt{2}$. The non-zero microwave detuning (*Δ* ≈ 1 kHz) causes the phase to accumulate between |↓⟩ and |↑⟩ during the hold, and the second pulse projects this onto the states |↓⟩ and |↑⟩. The populations of these states oscillate as a function of *T* with frequency *ν* = *Δ* − *Δα*_a.c._. For the entangling sequence of Fig. 3b, the microwaves are resonant with the transition and the pulse sequence prepares the pair state |*Φ*(*T*)⟩ (equation (1)).

When entangling molecules with direct microwave excitation, as shown in Fig. 3c, we set the Hann pulse such that the peak Rabi frequency *Ω*_0_ = 2.245 Hz. For the π/2 pulses used to read out the parity of the entangled state |*Ψ*^+^⟩, we use square pulses that drive the transition |↓⟩ → |↑⟩ with Rabi frequency *Ω* = 882(3) Hz.

### Experimental statistics

To obtain statistics, we repeat each experimental sequence many times. Data points in figures show the average state populations from these repeats and error bars show the 1*σ* binomial confidence intervals, calculated using the Jeffreys prior^70–72^ and are indicative of the number of repeats used to obtain each data point. Most data presented here are obtained by postselecting to ignore experimental runs in which molecule formation was unsuccessful or molecules were not detected in the states |↓⟩ or |↑⟩. We perform this postselection by using optical tweezers to map these cases onto distinct spatial configurations of atoms following the methods reported in ref. ^41^. Briefly, Rb atoms may be transferred into three distinct tweezer arrays: one flagging molecule-formation errors, one to detect |↑⟩ and one to detect |↓⟩. The Cs atom remains in the original tweezer array. At the end of each experimental sequence, we capture atomic fluorescence images to determine the atom locations. These are then used for shot-to-shot postselection, with a successful shot requiring recovery of both the Cs atom and the Rb atom in either the |↑⟩ or |↓⟩ array. For the data presented in Fig. 3a, in which we measure molecular lifetimes, we postselect to remove only detectable molecule-formation errors.

With postselection, we can obtain statistics for single- and two-molecule cases in a single set of experimental runs using the same sequence. For example, for each value of *Δ* in Fig. 2b, we repeat the experiment about 400 times. In 25% of runs, we successfully form and detect exactly one molecule in either the state |↓⟩ or |↑⟩. Therefore, each data point in the top panel represents about 100 samples of the binomial distribution, and the error bars are calculated accordingly. Likewise, in 7% of runs, we successfully form and detect exactly two molecules, and each data point in the bottom panel reflects about 30 samples.

### Simulations of the two-molecule system

To simulate the dynamics of the two-molecule system, we use the Python package QuTiP^73^ and model its time evolution with different microwave pulses and hold times.

The Hamiltonian that describes a pair of molecules interacting by the dipolar spin-exchange interaction in the presence of microwave coupling between |↓⟩ and |↑⟩ with a Rabi frequency *Ω* is^32^

2 $$H={H}_{{\rm{mol}}}^{(1)}\otimes {{\mathcal{I}}}^{(2)}+{{\mathcal{I}}}^{(1)}\otimes {H}_{{\rm{mol}}}^{(2)}+{H}_{{\rm{int}}}.$$

Here, ${H}_{{\rm{mol}}}^{(i)}=\frac{1}{2}h\varOmega ({\sigma }_{i}^{+}+{\sigma }_{i}^{-})-h{\varDelta }_{i}{| \uparrow \rangle }_{i}{\langle \uparrow | }_{i}$ is the single-particle Hamiltonian of molecule *i* and ${{\mathcal{I}}}^{(i)}$ is its identity operator. ${H}_{{\rm{int}}}=\frac{1}{2}hJ({\sigma }_{1}^{+}{\sigma }_{2}^{-}+{\sigma }_{2}^{+}{\sigma }_{1}^{-})$ is the interaction Hamiltonian, ${\sigma }_{i}^{+}\equiv {| \uparrow \rangle }_{i}{\langle \downarrow | }_{i}$ is the raising operator for molecule *i* and ${\sigma }_{i}^{-}\equiv {| \downarrow \rangle }_{i}{\langle \uparrow | }_{i}$ is the lowering operator for molecule *i*. *hJ* is the interaction strength and *Δ*_*i*_ is the microwave detuning from the transition ${| \downarrow \rangle }_{i}\to {| \uparrow \rangle }_{i}$. We allow for the fact that there may be a small difference $\delta \equiv \varDelta {\alpha }_{{\rm{a.c.}}}^{(2)}-\varDelta {\alpha }_{{\rm{a.c.}}}^{(1)}={\varDelta }_{1}-{\varDelta }_{2}$ in the differential a.c. Stark shifts of the molecules as they are in different traps. We generally denote pair states as $| ab\rangle \equiv {| a\rangle }_{1}\otimes {| b\rangle }_{2}$.

Our molecules are predominantly, but not exclusively, formed in the three-dimensional motional ground state^41^. This causes shot-to-shot noise in *J* as the separation averaged over the molecular wavefunctions varies. We incorporate this in our model with a Monte Carlo method: the dynamics are averaged over 200 iterations for which we assume that shot-to-shot noise in *J* is such that, in each experimental iteration, *J* is sampled from a Gaussian distribution with mean ⟨*J*⟩ and standard deviation *σ*_*J*_.

#### Eigenstates in the absence of microwaves

Equation (2) gives the Hamiltonian *H* that describes our system of two interacting molecules. In the absence of microwave radiation, *H* simplifies to

3 $${H}_{0}=\frac{h}{2}\left(\begin{array}{cccc}0 & 0 & 0 & 0\\ 0 & \delta  & J & 0\\ 0 & J & -\delta  & 0\\ 0 & 0 & 0 & 0\end{array}\right),$$

in the basis {|↓↓⟩, |↓↑⟩, |↑↓⟩, |↑↑⟩}. The eigenstates of *H*_0_ are |↓↓⟩, |↑↑⟩,

4 $$| {\widetilde{\varPsi }}^{+}\rangle ={N}_{+}\left(\begin{array}{c}0\\ J\\ \sqrt{{J}^{2}+{\delta }^{2}}-\delta \\ 0\end{array}\right)\,{\rm{and}}$$

5 $$| {\widetilde{\varPsi }}^{-}\rangle ={N}_{-}\left(\begin{array}{c}0\\ \sqrt{{J}^{2}+{\delta }^{2}}-\delta \\ -J\\ 0\end{array}\right),$$

where *N*_±_ are normalization constants.

In the main text, we consider the limit of strong interactions (that is, ∣*J*∣/∣*δ*∣ → *∞*), where $| {\widetilde{\varPsi }}^{\pm }\rangle \to | {\varPsi }^{\pm }\rangle \equiv (| \downarrow \uparrow \rangle \pm | \uparrow \downarrow \rangle )\sqrt{2}$. However, the non-zero value of *δ* in our experiment gives rise to eigenstates that are slightly asymmetric. The eigenstates of our system, taking *J* = 5.20 Hz and *δ* = 220 mHz, are $| {\widetilde{\varPsi }}^{+}\rangle =0.722| \downarrow \uparrow \rangle +0.692| \uparrow \downarrow \rangle$ and $| {\widetilde{\varPsi }}^{-}\rangle =0.692| \downarrow \uparrow \rangle -0.722| \uparrow \downarrow \rangle$, in which the coefficients are given to three significant figures.

In Fig. 2b (bottom), we show microwave spectroscopy in which we drive the transition |↓↓⟩ → |*Ψ*^*+*^⟩. The asymmetry in the probability amplitudes |↓↑⟩ and |↑↓⟩ in |*Ψ*^*+*^⟩ is the reason why we measure a slightly higher population in the state |↓↑⟩ than in the state |↑↓⟩. This has only a slight effect on the achieved entanglement fidelity, the dominant limitations of which are the non-zero value of *σ*_*J*_ and leakage errors caused by Raman scattering from the tweezer light.

#### Design of direct-entanglement pulse

For the demonstration of the two-molecule microwave gate shown in Fig. 3c, we use a pulse with a simple shape. We choose the parameters of this pulse using our Monte Carlo model with the parameters fitted from the data in Fig. 2.

First, we model and optimize the pulse assuming that there is no noise in *J*. We take *J* to be equal to the measured value of ⟨*J*⟩ (5.20 Hz) and consider three simple pulse shapes: a square pulse (*Ω*(*t*) = *Ω*_0_ for 0 < *t* < *τ*, 0 otherwise), a Hann pulse (*Ω*(*t*) = *Ω*_0_ sin^2^(π*t*/*τ*)) and a Blackman–Harris pulse (*Ω*(*t*) = *Ω*_0_ [*a*_0_ − *a*_1_cos(2π*t*/*τ*) + *a*_2_cos(4π*t*/*τ*) − *a*_3_cos(6π*t*/*τ*)] for *a*_0_ = 0.35875, *a*_1_ = 0.48829, *a*_2_ = 0.14128 and *a*_3_ = 0.01168). Here, *Ω*(*t*) is the Rabi frequency that we drive the single-molecule transition |↓⟩ → |↑⟩, *Ω*_0_ is the peak Rabi frequency and *τ* is the pulse duration. For each pulse shape, we vary *Ω*_0_ and calculate *P*_*↓↑*_ + *P*_*↑↓*_ as a function of *τ* and the microwave detuning *Δ* (Extended Data Fig. 3, inset). *P*_*↓↑*_ + *P*_*↑↓*_ is a good proxy for the fidelity of the entangling gate because pairs that are not entangled preferentially occupy the states |↓↓⟩  and |↑↑⟩. This gives an optimum value of *τ* and *Δ* for each value of *Ω*_0_, with an associated maximum (*P*_↓↑_ + *P*_↑↓_)_max_. We show the behaviour of (*P*_↓↑_ + *P*_↑↓_)_max_ on *τ* in Extended Data Fig. 3 (top); a longer pulse duration generally allows higher fidelity entanglement because a smaller Rabi frequency can be used to minimize off-resonant excitation to |↑↑⟩.

We now consider fluctuations in *J*. With the optimum pulse parameters obtained above, we use our Monte Carlo model to recalculate (*P*_↓↑_ + *P*_↑↓_)_max_ when *σ*_*J*_ = 1 Hz (Extended Data Fig. 3, bottom). The effect of *σ*_*J*_ is to favour larger Rabi frequencies (that is, smaller *τ*), which spectrally broaden the excitation feature. We expect that, out of the pulse shapes considered, a Hann pulse will achieve the highest (*P*_↓↑_ + *P*_↑↓_)_max_. The corresponding pulse parameters are *τ* = 328 ms, *Δ* = 3.069 Hz and *Ω*_0_ = 2.245 Hz; we use these for the experiments presented in Fig. 3.

### Expected interaction strength

Here we consider the strength of the spin-exchange interaction between the molecular pair states |↓↑⟩ and |↑↓⟩. First, we consider the case in which the molecules can be treated as point particles with zero temperature. Then, we estimate the effect that the non-zero temperature and wavefunction size have on this interaction strength.

#### Point-particle and zero-temperature case

The strength of the dipole–dipole interaction between the states |↓↑⟩ and |↑↓⟩ is^32^

6 $$J=-\frac{1}{h}\frac{1-3{\cos }^{2}\theta }{| {{\bf{r}}}_{1}-{{\bf{r}}}_{2}\,{| }^{3}}\frac{{d}_{\downarrow \uparrow }^{2}}{4{\rm{\pi }}{\varepsilon }_{0}}.$$

Here, **r**_*i*_ is the position vector of molecule *i* and *θ* is the angle between the quantization axis and the intermolecular vector. *ε*_0_ is the vacuum permittivity. ${d}_{\downarrow \uparrow }\equiv \langle \uparrow | {\widehat{d}}_{1}| \downarrow \rangle$ is the relevant matrix element for the dipole operator ${\widehat{d}}_{1}$ that corresponds to the *σ*^+^ transition that we use. At zero electric field, ${d}_{\downarrow \uparrow }=d/\sqrt{3}$, where *d* = 1.225(11) D is the RbCs molecule-frame electric dipole moment^74^.

For all experiments, the intermolecular axis is parallel to the quantization axis (that is, *θ* = 0). We apply no external electric fields and assume that the stray electric field is negligible. For the experiment presented in Fig. 1, we prepare molecules at a separation |**r**_1_ − **r**_2_| = 8.6(2) μm. Therefore, if the molecules were point particles pinned to the centre of their respective optical tweezer, we would expect *J* = 0.24(1) Hz. For this reason, we limit the interrogation time for this measurement to *T* ≲ 2 s so that these interactions are insignificant. Likewise, for the experiments presented in Figs. 2 and 3, |**r**_**1**_ − **r**_**2**_| = 2.78(5) μm giving *J* = 7.0(4) Hz. In both cases, the uncertainty in *J* reflects the uncertainty in the molecular separation.

#### Effect of motional excitation

We fit the microwave spectroscopy shown in Fig. 2 with a Monte Carlo model, in which *J* is sampled from a normal distribution for every iteration of the experiment. Using this model, we extract the mean ⟨*J*⟩ = 5.20(5) Hz and standard deviation *σ*_*J*_ = 1.0(1) Hz.

We expect that motional excitation of the molecules causes the reduction in ⟨*J*⟩ from the expected value and is the dominant contribution to *σ*_*J*_. To estimate the magnitude of this effect, we numerically calculate the matrix elements

7 $$\bar{J}({{\bf{n}}}_{1};{{\bf{n}}}_{2})=-\frac{1}{h}\frac{{d}_{\downarrow \uparrow }^{2}}{4{\rm{\pi }}{\varepsilon }_{0}}\left\langle {{\bf{n}}}_{1}{{\bf{n}}}_{2}\left|\frac{1-3{\cos }^{2}\theta }{{| {{\bf{r}}}_{1}-{{\bf{r}}}_{2}| }^{3}}\right|{{\bf{n}}}_{1}{{\bf{n}}}_{2}\right\rangle ,$$

where $| {{\bf{n}}}_{i}\rangle \equiv | {n}_{x}^{i},{n}_{y}^{i},{n}_{z}^{i}\rangle$ is the three-dimensional wavefunction for molecule *i*, labelled by the number of motional quanta in each of the three directions. Here, we define the *x*-axis as the quantization axis, the *y*-axis as the other radial axis of the tweezers and the *z*-axis as the direction of tweezer-light propagation, as shown in Extended Data Fig. 1a. We assume that the trapping potential is harmonic and the three axes are separable such that

8 $$\langle {{\bf{r}}}_{i}| {{\bf{n}}}_{i}\rangle =\prod _{r\in \{{x}_{i},{y}_{i},{z}_{i}\}}C({n}_{r}){H}_{{n}_{r}}(r/{\beta }_{r}){{\rm{e}}}^{-{r}^{2}/2{\beta }^{2}},$$

where ${H}_{{n}_{r}}$ are the Hermite polynomials and the index *r* runs over the three separable axes. ${\beta }_{r}=2{\rm{\pi }}\sqrt{m{\nu }_{r}/h}$ and *ν*_*r*_ are the confinement length and trap frequency along the *r*-axis, respectively, and the normalization constant $C({n}_{r})=1/\sqrt{({2}^{{n}_{r}}{n}_{r}!{\beta }_{r}{{\rm{\pi }}}^{1/2})}$.

**Extended Data Fig. 2:**
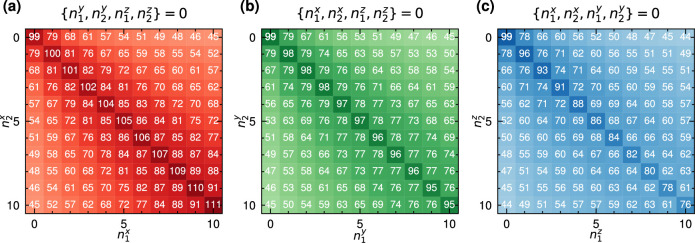
Calculated matrix elements $\bar{J}$. Matrix elements for molecules motionally excited along the (a) *x*- (b) *y*- and (c) *z*-axes. The modal intermolecular separation is 2.78 *μ*m along the *x*-axis. The colours and numbers label the value of $\bar{J}\,/J$ (in percent) where *J* = 7.0 Hz is the expected value for a point-particle at zero-temperature. Source Data

Extended Data Fig. 2 shows calculations of selected values of $\bar{J}$. In general, $\bar{J}$ is a six-dimensional matrix; we show the three slices of this matrix in which the motional quanta of the molecules along one axis is varied, whereas there is no motional excitation along the other axes. For this calculation, the separation between the most likely positions of the molecules is 2.78 μm along the *x*-axis. The molecules are trapped in tweezers of waist 1.76 μm and intensity 4.5 kW cm^−2^. We neglect the effect of the tweezer confining the first molecule on the second molecule (and vice versa) and assume that fluctuations in the relative positions of the tweezers are negligible as they are formed from a common source^60^. We take the polarizability of the molecules at the magic wavelength to be $720\times 4{\rm{\pi }}{\varepsilon }_{0}{a}_{0}^{3}$ (ref. ^11^) such that the trap frequencies are *ν*_*x*_ = *ν*_*y*_ = 3.0 kHz and *ν*_*z*_ = 0.4 kHz.

We estimate that 58(6)% of molecules formed in the 1,066 nm array occupy the three-dimensional motional ground state^41,75^. Furthermore, we expect that most of the motionally excited molecules have just one motional quantum. Therefore, the most likely scenario is that, when a pair of molecules is formed, one occupies the motional ground state and the other has one motional quantum. Assuming negligible heating as the molecules are transferred to the magic tweezers, the relevant matrix element $\bar{J}\approx 5.5(3)\,{\rm{Hz}}$. This is approximately equal to our measured value of ⟨*J*⟩, and the stochastic occupancy of the motional states will give rise to *σ*_*J*_.

In future, we expect that moving to more confining traps (for example, by trapping the molecules in an optical lattice) will allow smaller separations and reduce the wavefunction spread, leading to an increase in ⟨*J*⟩ and a reduction in *σ*_*J*_. We note that *σ*_*J*_ could also be reduced by increasing the fraction of molecules that occupy the three-dimensional motional ground state by reducing atomic heating before association^62,75^.

## Online content

Any methods, additional references, Nature Portfolio reporting summaries, source data, extended data, supplementary information, acknowledgements, peer review information; details of author contributions and competing interests; and statements of data and code availability are available at 10.1038/s41586-024-08365-1.

## Source data

Source Data Fig. 1

Source Data Fig. 2

Source Data Fig. 3

Source Data Extended Data Fig. 2

**Extended Data Fig. 3:**
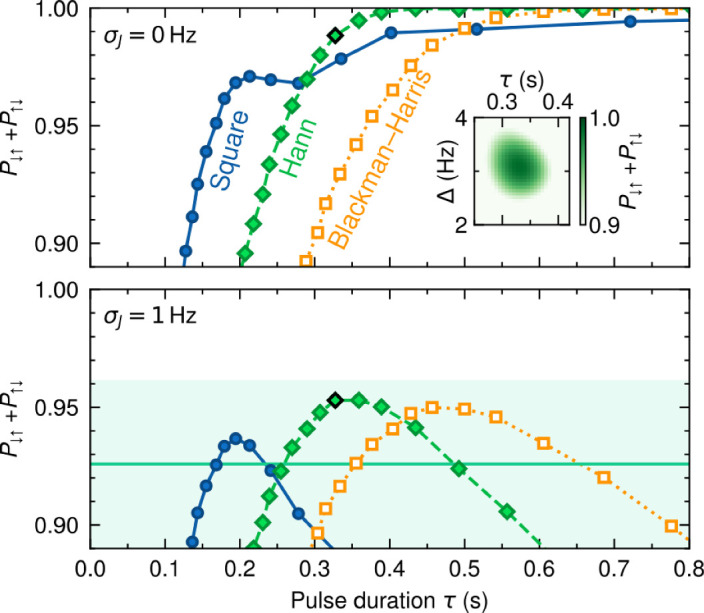
Optimisation of a microwave pulse for direct entanglement. Top: Predicted state populations *P*_*↓↑*_ + *P*_*↑↓*_ for different pulse durations *τ* when there is no noise in *J*. The peak Rabi frequency *Ω*_0_ is set for each value of *τ* to achieve the maximum value of *P*_*↓↑*_ + *P*_*↑↓*_. The inset shows *P*_*↓↑*_ + *P*_*↑↓*_ as a function of *Δ* and *τ* when *Ω*_0_ = 2.245 Hz and a Hann pulse is used. The peak value ${({P}_{\downarrow \uparrow }+{P}_{\uparrow \downarrow })}_{\max }$ and the optimum value of *τ* correspond to the highlighted point in the main figure. This is simulated for various *Ω*_0_ to find the optimum parameters. Bottom: As above, but when *σ*_*J*_ = 1 Hz. The highlighted point corresponds to the pulse parameters used when taking the data presented in Fig. 3. The horizontal line shows the peak value of *P*_*↓↑*_ + *P*_*↑↓*_ measured in Fig. 3(c) and the shaded region shows the 1*σ* confidence interval. Source Data

Source Data Extended Data Fig. 3

## Data Availability

The data that support the findings of this study are available at 10.15128/r1bv73c047f. Source data are provided with this paper.
